# Anti-inflammatory consequences of bile acid accumulation in virus-infected bile duct ligated mice

**DOI:** 10.1371/journal.pone.0199863

**Published:** 2018-06-28

**Authors:** Stephanie Rattay, Dirk Graf, Andreas Kislat, Bernhard Homey, Diran Herebian, Dieter Häussinger, Hartmut Hengel, Albert Zimmermann, Anna-Kathrin Schupp

**Affiliations:** 1 Institute of Virology, Heinrich-Heine-University, University Hospital, Duesseldorf, Germany; 2 Institute of Clinical Chemistry and Clinical Pharmacology, University Hospital, Bonn, Germany; 3 Department of Gastroenterology, Hepatology and Infectious Diseases, Heinrich-Heine-University, University Hospital, Duesseldorf, Germany; 4 Department of Dermatology, Heinrich-Heine-University, University Hospital, Duesseldorf, Germany; 5 Department of General Pediatrics, Neonatology and Pediatric Cardiology, Heinrich-Heine-University, University Hospital, Duesseldorf, Germany; 6 Institute of Virology, Medical Center, Albert-Ludwigs-University, Freiburg, Germany; 7 Department for Medical Microbiology and Hygiene, Institute of Virology, Faculty of Medicine, Albert-Ludwigs-University, Freiburg, Germany; University of St Andrews, UNITED KINGDOM

## Abstract

Cholestatic patients exhibiting high bile acid serum levels were reported to be more susceptible to bacterial and viral infections. Animal studies in bile duct ligated (BDL) mice suggest that cholestasis leads to an aggravation of hepatic bacterial infections. We have investigated the impact of cholestasis on mouse cytomegalovirus (MCMV)-induced immune responses and viral replication. While MCMV did not aggravate BDL-induced liver damage, BDL markedly reduced MCMV-triggered chemokine expression and immune cell recruitment to the liver. MCMV-infected BDL mice showed diminished trafficking of Ly6C^+^/F4/80^+^ myeloid cells and NK1.1^+^ NK cells to the liver compared to MCMV infected control mice. Moreover, virus-driven expression of CCL7, CCL12, CXCL9 and CXCL10 was clearly impaired in BDL- compared to sham-operated mice. Furthermore, production of the anti-inflammatory cytokine IL-10 was massively augmented in infected BDL mice. In contrast, intra- and extrahepatic virus replication was unaltered in BDL-MCMV mice when compared to sham-MCMV mice. Cholestasis in the BDL model severely impaired pathogen-induced chemokine expression in the liver affecting CCR2- and CXCR3-dependent cell trafficking. Cholestasis resulted in reduced recruitment of inflammatory monocytes and NK cells to the liver.

## Introduction

Cholestatic conditions, i.e. elevation of serum bile acid levels arise when bile formation, secretion or flow from the liver to the gut is disrupted. This can be induced by metabolic conditions (e.g. drug induced hepatotoxicity, autoimmune liver diseases, viral infections of the liver) or mechanical obstruction of bile ducts (e.g. by tumors or gallstones). Bile acids represent the major content of bile and are synthesized from cholesterol in hepatocytes, the main epithelial cell type of the liver. A key function of bile acids is to support fatty acids uptake into the gut. Beyond that, bile acids act as signal molecules thereby influencing cellular glucose and lipid metabolism, growth and gene expression [1,2]. Moreover, it was shown that high concentrations of bile acids, as manifested in cholestatic patients, induce hepatitis B and C virus replication [3–5]. Bile acid accumulation is associated with impaired function of monocytes, macrophages, NK cells and T cells *in vitro*, resulting in diminished cytokine expression of immune cells like macrophages and DCs [2,6,7]. In addition, bile acids influence cellular immunity by inhibiting interferon (IFN)-α und interleukin (IL)-6 signaling in hepatocytes [8,9]. Consequently, cholestatic patients exhibit higher susceptibility to viral and bacterial infections and have a high infection associated mortality rate [10].

Bile duct ligation (BDL) is an established and widely used experimental rodent model to study the generation and pathogenic consequences of cholestasis and fibrosis in a standardized way *in vivo* [11]. BDL-operated animals infected by portal venous injection with *Escherichia coli* exhibit a higher mortality rate and increased bacterial growth compared to sham-operated animals and generate higher IL-10 expression levels [12]. IL-10 has a rather anti-inflammatory function and negatively impacts macrophage function and expression of pro-inflammatory cytokines. Accordingly, a study utilizing human lipopolysaccharide (LPS)-activated macrophages demonstrated impaired pro-inflammatory cytokine expression after bile acid treatment while IL-10 expression was stable [7].

Cytomegaloviruses (CMV) are hepatotropic members of the *β-herpesvirinae* family, which persist lifelong in infected individuals during alternating phases of productive replication and latency. Primary CMV infection leads to virus dissemination and replication in different organs. Within the liver the major target cells for CMV infections are hepatocytes, liver sinusoidal endothelial cells, biliary epithelial cells and Kupffer cells [13,14]. While human cytomegalovirus (HCMV; human herpesvirus 5) infections in immuno-competent individuals normally proceed subclinically, in immuno-compromised patients CMV infections often cause overt disease, including hepatitis and cholestasis [15]. CMV related cholestasis is common in liver transplant recipients since 30–50% of all patients show signs of CMV infections [16]. Due to the fact that all CMVs have developed a strict species-specificity during co-evolution with their host, *in vivo* experiments with HCMV in animal models are not possible. Thus, the homologous mouse CMV (MCMV, Murid herpesvirus 1), which infects *Mus musculus* as its natural host, is widely utilized to analyze basic principles of virology and immunology in a natural host-pathogen situation. HCMV and MCMV exhibit a similar cell- and organ-tropism and cause analogous diseases [17]. CMV infection of cells is recognized by cellular pattern recognition receptors e.g. *toll-like receptors* and cytoplasmic sensors [18–20]. Induction of different signaling pathways leads to expression of a broad spectrum of cytokines including interferons and chemokines, which in turn activate the immune response. Immune cell infiltration is a result of cytokine/chemokine expression by tissue cells and activated resident immune cells. As cytokines play a significant role in immune induction, impaired immune cell function under cholestatic conditions could be associated with modified cytokine expression profiles.

Previous work investigated the effect of BDL-induced cholestasis on bacterial infection and the antibacterial immune response [12,21]. This study was aimed to investigate the consequences of cholestasis on viral infection and *vice versa*. To our knowledge, this study describes for the first time the impact of cholestasis on antiviral immune responses and viral replication in the BDL model.

## Results

### MCMV infection does not aggravate BDL-induced liver damage

Cholestatic liver injury can be explained either by direct bile acid toxicity or by inflammatory liver injury [22]. To investigate the impact of MCMV infection on BDL-induced liver damage sham-operated (sham) or BDL C57/BL6 mice were either mock treated (PBS injection) or infected intraperitoneally with MCMV-luc (2x10^5^ PFU) directly after operation. Blood plasma was harvested to determine *glutamate pyruvate transaminase (GPT)* activity as well as bile acid concentration and composition. Liver damage was determined by plasma GPT activity which was strongly elevated in BDL and BDL-MCMV animals compared to sham and sham-MCMV mice (Table 1). GPT activity was comparable in BDL-MCMV and BDL animals. Blood plasma was also used to measure bile acid levels. Quantification of plasma bile acids revealed a significant increase of total bile acids concentration in BDL or BDL-MCMV compared to sham mice. MCMV infection slightly elevated total bile acid levels compared to sham treated mice (Table 1), but this effect was not significant. Since the main content of bile acids in mice was taurine-conjugated, the plasma levels of taurine-conjugated bile acids in total and specific group members as taurochenodeoxycholic acid (TCDC) and taurocholic acid (TC) were quantified. TCDC and TC plasma concentrations in BDL and BDL-MCMV mice were significantly higher compared to sham mice (Table 1). The group of glycine-conjugated bile acids was also significantly elevated.

**Table 1:** BDL leads to increased bile acid concentrations in blood plasma.

Clinical parameter	sham	BDL	sham-MCMV	BDL-MCMV
GPT [IU/L]	12.4 +/- 7.6	3186 +/- 775	71+/- 132	2031 +/- 1219
muricholic acids [μmol/l]	1.2 +/- 0.4	1880 +/- 165	3.7 +/- 2.5	2403 +/- 2108
taurine-conjugated [μmol/l]	1.2 +/- 0.4	1880 +/- 165	4.0 +/- 2.9	2793 +/- 1539
glycine conjugated [μmol/l]	0.1 +/-0.0	44.2 +/-17.0	0.4 +/- 0.4	39.6 +/- 33.2
TCDC [μmol/l]	0.02 +/-0.01	1.8 +/- 0.7	0.1 +/- 0.1	1.7 +/- 1.9
TC [μmol/l]	0.6 +/- 0.2	1399+/- 243	2.0 +/- 1.6	1768 +/- 1172

Sham- or BDL-operated mice were mock treated or infected with MCMV-luc (2x10^5^ PFU/ml). 72 hpi blood of the *vena cava* was harvested and plasma bile acid concentrations were analyzed by UPLC-MS/MS. The concentrations of unconjugated, taurine-conjugated and glycine-conjugated forms of α-/β-/ω-muricholic acid, chenodeoxycholic acid, cholic acid, deoxycholic acid, hyodeoxycholic acid, lithocholic acid, hyocholic acid and ursocholic acid were determined. The group of muricholic acids (MCAs) includes unconjugated, taurine-conjugated and glycine-conjugated forms of α-MCA, β-MCA and ω-MCA. The groups of taurine- or glycine-conjugated bile acids contain conjugates of α-/β-/ω-muricholic acid, chenodeoxycholic acid, cholic acid, deoxycholic acid, lithocholic acid, hyodeoxycholic acid, hyocholic acid and ursodeoxycholic acid. Plasma GPT activity was determined by spectroscopy. Depicted are the mean values ± SD (Sham n = 5; BDL n = 4; sham-MCMV n = 9; BDL-MCMV n = 9). Statistical significance was determined with Mann-Whitney-U-tests.

Clinically, all BDL-operated animals showed signs of jaundice at skin and liver after 72 h. However, no overt differences in physical condition and survival times were observed comparing BDL and BDL-MCMV mice. Overall, BDL leads to a markedly increase of bile acid levels and an overt liver damage documented by high GPT levels. However, simultaneous MCMV infection did not worsen clinical parameters and outcome substantially.

### Recruitment of immune cells to MCMV-infected livers is reduced by BDL

**Fig 1:**
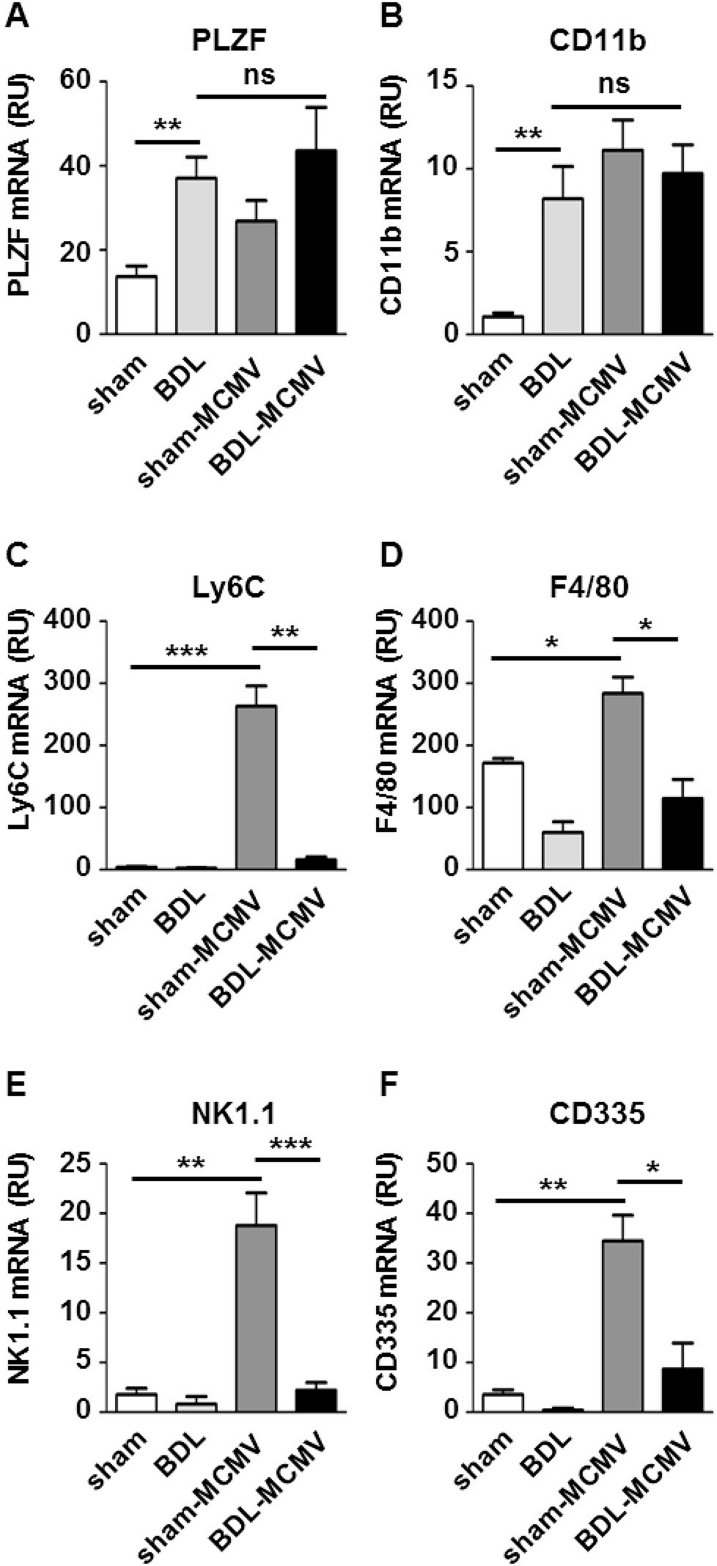
BDL alters the immune cell profile of MCMVinfected livers. Sham- or BDL-operated mice were either mock treated or infected with MCMV-luc (2x10^5^ PFU/ml) and 72 hpi organs were harvested. Total RNA of liver tissue was prepared and cell marker mRNA expression was analyzed by qPCR using specific TaqMan primers and probes. Relative gene expression was calculated by the ΔΔCt method using RPII or SDHA as housekeeping gene. Depicted are the mean values ± SEM of (A) PLZF, (B) CD11b, (C) Ly6C, (D) F4/80, (E) NK1.1 and (F) CD335 from the indicated groups of mice (sham n = 5; BDL n = 4; sham MCMV n = 9; BDL MCMV n = 9). Statistical significance was calculated with Mann-Whitney-U-tests (***p< 0.001, **p< 0.01, *p< 0.05, ns: not significant).

To monitor immune cell infiltration into the liver, we prepared total RNA of liver tissue of sham or BDL-operated (un)infected mice and quantified expression of innate immune cell markers by qPCR [23]. The marker panel used included PLZF (NKT cells), CD11b (neutrophils), Ly6C (inflammatory monocytes), F4/80 (macrophages), NK1.1 and CD335 (NK cells). To identify BDL-induced differences in immune cell marker expression we performed statistical analysis by Kruskal-Wallis tests comparing expression levels of sham animals to BDL animals. Expression of PLZF (Fig 1A) and CD11b (Fig 1B) was significantly increased by BDL. Enhanced expression of CD11b in BDL-operated mice may reflect the well-known accumulation of neutrophils after BDL [22]. The influence of MCMV infection on BDL-dependent immune cell infiltration was determined by statistical analysis comparing BDL animals to MCMV-infected BDL mice. PLZF and CD11b expression was also increased in MCMV-infected sham mice (Fig 1A and 1B). However, BDL and simultaneous MCMV infection did not result in significant changes compared to BDL-dependent marker expression.

MCMV infection is known to result in recruitment of macrophages, inflammatory monocytes and NK cells to the infected liver [24,25]. To identify MCMV-induced alterations in immune cell marker expression we performed statistical analysis by Kruskal-Wallis tests comparing expression levels of sham animals to sham-MCMV animals. The influence of BDL on virus-dependent immune cell infiltration was determined by statistical analysis comparing MCMV-infected sham animals to MCMV-infected BDL mice. The monocyte/macrophage markers Ly6C and F4/80 were significantly increased after MCMV-infection (Fig 1C and 1D). Importantly, both markers were clearly decreased in MCMV-infected BDL-mice compared to sham-operated infected animals indicating that BDL might impair the MCMV induced immune cell migration to the liver. The impact of cholestasis on MCMV-dependent NK-cell recruitment was determined by qPCR analysis of the NK-cell markers NK1.1 (Fig 1E) and CD335 (Fig 1F). As expected, both markers were strongly increased in sham-MCMV animals. Again, the expression of NK1.1 and CD335 was significantly reduced in BDL-MCMV compared to sham-MCMV mice. CD335 also appeared slightly increased comparing the BDL and BDL-MCMV group, however, this was due to a higher variation of the BDL-MCMV values and is not statistically significant. In summary, these results demonstrate that cholestasis in BDL animals might impair the MCMV-induced immune cell migration to the liver.

**Fig 2:**
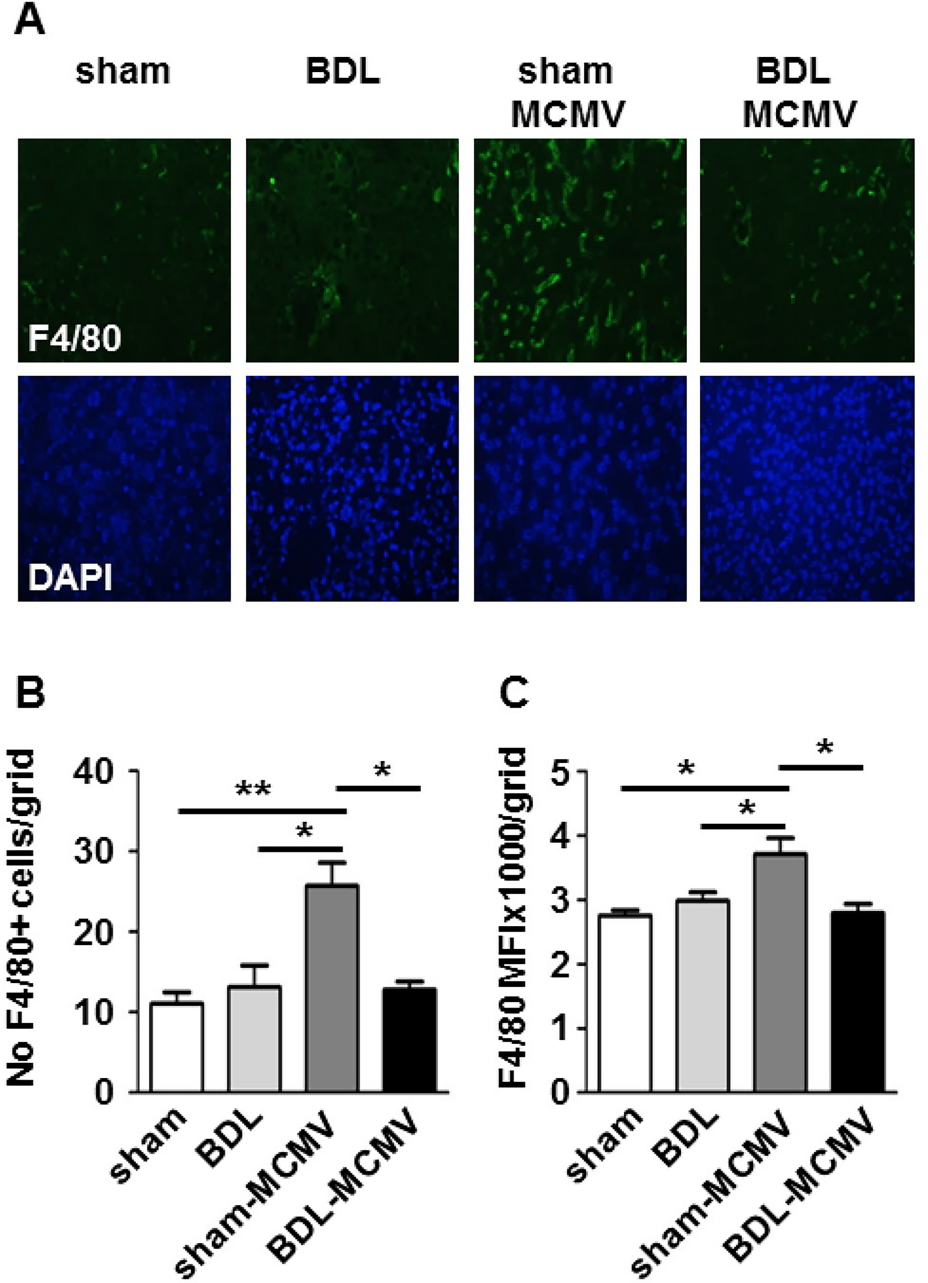
BDL reduces the recruitment of immune cells to MCMV-infected livers. Sham- or BDL-operated mice were mock treated or infected with MCMV-luc and 72 hpi livers were harvested. (A) Liver sections were prepared using a cryotome followed by immunohistochemistry staining with specific antibodies against the macrophage marker F4/80. Nuclei were stained with DAPI. Depicted are representative pictures of each group. F4/80 expression was quantified by (B) counting F4/80^+^ cells in liver sections and (C) determination of the mean fluorescence intensity of stained liver sections. Depicted are the mean values ± SEM from the indicated groups of mice (sham n = 5; BDL n = 4; sham-MCMV n = 9; BDL-MCMV n = 9). Statistical significance was calculated with Mann-Whitney-U-tests (**p< 0.01, *p< 0.05).

To validate these results we traced MCMV-induced immune cell infiltration under cholestasis by immunofluorescence microscopy. F4/80 was used as a macrophage marker in liver sections. As expected, immunofluorescence analysis of sham-MCMV liver sections revealed increased abundance of F4/80^+^ cells compared to sham and BDL liver sections (Fig 2A). To quantify these results we determined the frequencies of F4/80^+^ cells (Fig 2B) and the mean fluorescence intensities (MFI, Fig 2C) under the different conditions. The results indicated a clearly increased frequency of F4/80^+^ cells in the livers of MCMV-infected mice. Importantly, no comparable increase of F4/80^+^ cells was observed in liver sections of MCMV-infected BDL mice.

Taken together, these data indicate an impaired MCMV-induced infiltration of macrophages and NK cells under cholestasis.

### MCMV-induced chemokine expression is diminished by BDL

**Fig 3:**
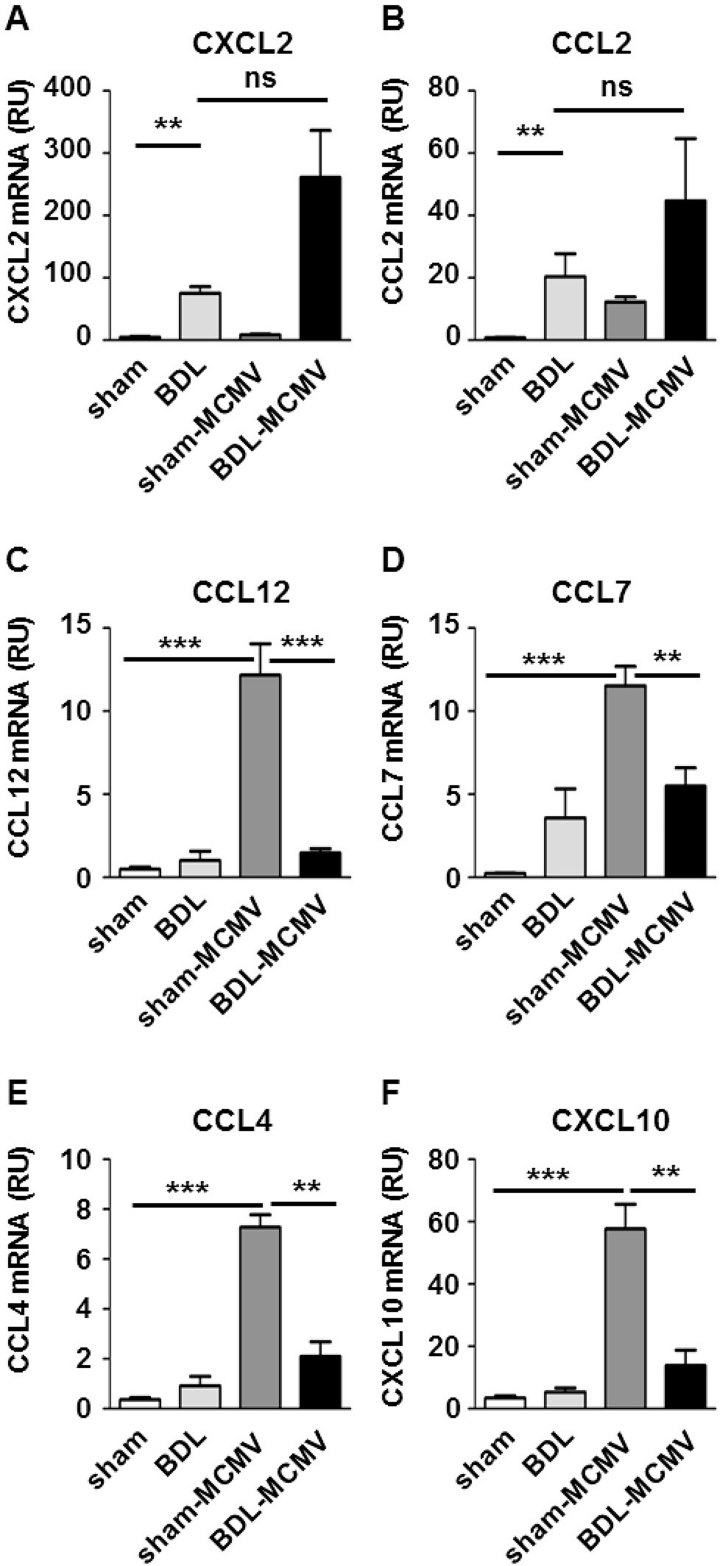
BDL diminishes MCMV-induced chemokine induction. Sham or BDL operated mice were either mock treated or infected with MCMV-luc (2x10^5^ PFU/ml) and 72 hpi organs were harvested. Total RNA of liver tissue was prepared and chemokine mRNA expression was analyzed by qPCR using specific primers or TaqMan primers and probes. Relative gene expression was calculated by the ΔΔCt method using ActB or SDHA as housekeeping gene. Depicted are the mean values ± SEM of (A) *Cxcl2*, (B) *Ccl2*, (C) *Ccl12*, (D) *Ccl7*, (E) *Ccl4* and (F) *Cxcl10* from the indicated groups of mice (sham n = 5; BDL n = 4; sham-MCMV n = 9; BDL-MCMV n = 9). Statistical significance was calculated with Mann-Whitney-U-tests (***p< 0.001, **p< 0.01, *p< 0.05, ns: not significant).

Since immune cell infiltration is mainly regulated by pro- and anti-inflammatory chemokines and cytokines, expression levels of chemokines and cytokines were measured. Bile acids have been described to directly induce chemokine expression in hepatocytes [26]. However, earlier reports demonstrated that BDL results in reduced pro-inflammatory cytokine expression and impaired bacterial clearance [12,27]. To investigate the impact of BDL on virus driven chemokine and cytokine induction we determined the expression levels of 19 chemokines (*Ccl2*, *Ccl3*, *Ccl4*, *Ccl5*, *Ccl7*, *Ccl8*, *Ccl9*, *Ccl11*, *Ccl12*, *Ccl17*,*Ccl20*, *Cxcl1*, *Cxcl2*, *Cxcl9*, *Cxcl10*, *Cxcl11*, *Cxcl12*, *Cxcl14 and Cx3cl1)* in livers of (un)infected sham or BDL mice by qPCR 72 hpi. Statistical significance was determined by Kruskal-Wallis testing. BDL resulted in statistically significant up-regulation of *Cxcl2* (Fig 3A), *Ccl2* (Fig 3B) and *Cxcl14* (S1 Fig). The simultaneous infection with MCMV did not significantly modify the BDL-driven chemokine induction. Hence, no synergistic chemokine induction by BDL and MCMV infection could be observed.

The infection of sham-operated mice with MCMV resulted in the induction of a different chemokine subset containing *Ccl12* (Fig 3C), *Ccl7* (Fig 3D), *Ccl4* (Fig 3E) and *Cxcl10* (Fig 3F). Further MCMV-regulated chemokines were *Ccl5*, *Ccl8 and Cxcl9* (S1 Fig). Remarkably, the combination of BDL and MCMV infection resulted in significantly diminished transcription of these chemokines compared to sham-MCMV mice (Fig 3C–3F). These results indicate that accumulation of bile acids in the liver leads to a significantly diminished expression of certain MCMV-induced chemokines. Of note, most BDL-dependent changes in virus-driven chemokine induction are detectable already 24 hpi indicating that cholestasis severely perturbs early MCMV-dependent chemokine induction (S2 Fig). Importantly, BDL-induced reduction of chemokine expression could interfere with two major pathways of antiviral immune cell recruitment. CCL7, CCL8 and CCL12 are CCR2 ligands involved in monocyte recruitment [28] whereas CXCL9 and CXCL10 are CXCR3 ligands known to contribute to T cell migration to the MCMV-infected liver [29] but also harbor the potential to attract NK cells [30].

### BDL regulates MCMV-induced cytokines

Liver-specific expression of the cytokines *Tnf-α*, *Ifn-β*, *Ifn-γ*, *Il-12*, *Il-10* and *Il-6* was analyzed 72 h post treatment by qPCR. IFN-γ, IFN-β and IL-12 are described to interfere with MCMV replication while TNF-α augments the antiviral effects of IFN-γ [31–33]. IL-10 is induced by MCMV infection and has immune regulatory functions blunting the expression of anti-cytomegaloviral pro-inflammatory signals in the liver [34]. IL-6 is induced by acute CMV infection of fibroblasts and is associated with angiogenesis [35].

**Fig 4:**
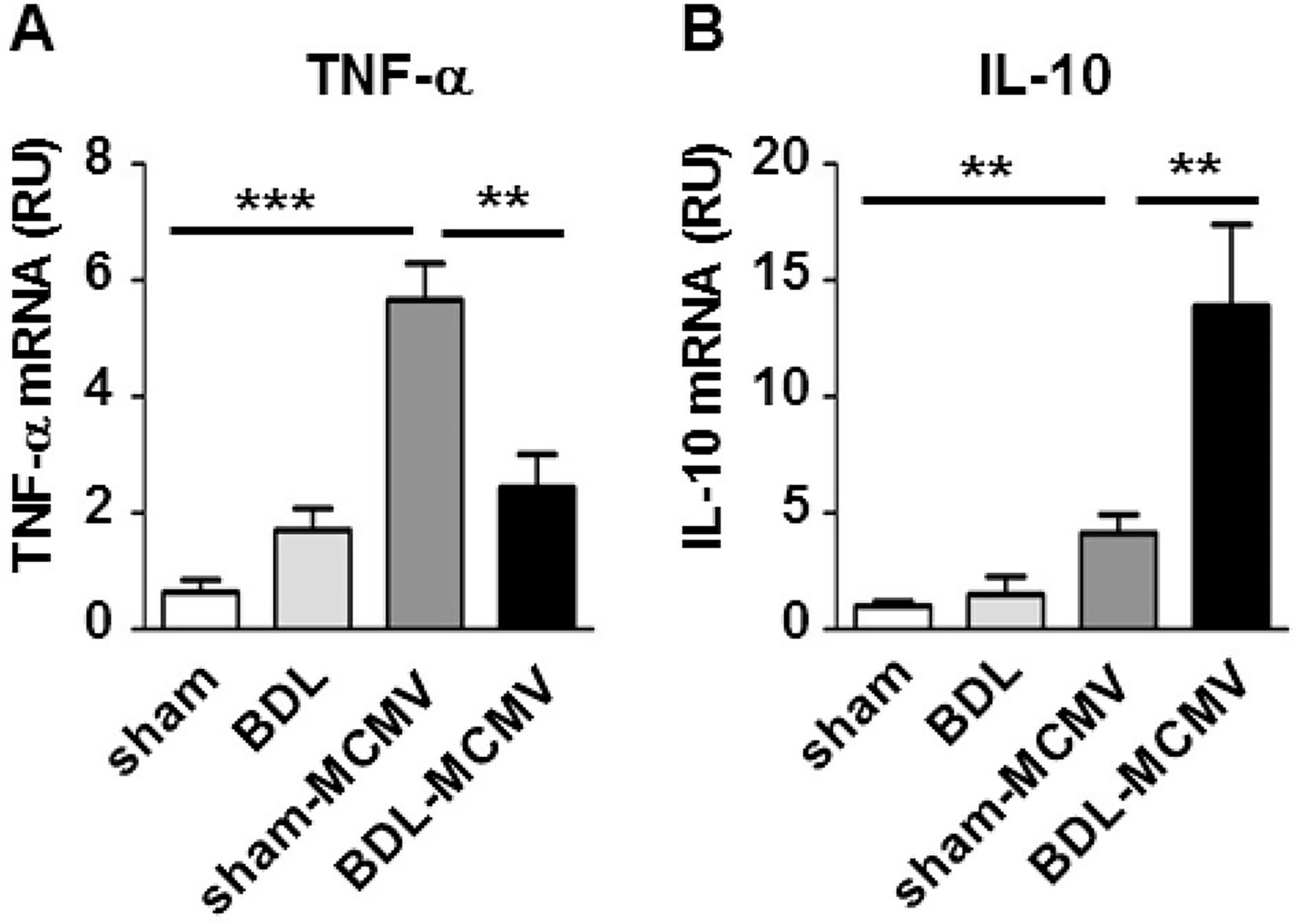
BDL modifies *Tnf-α* and *Il-10* expression in the liver. Organs of mock or MCMV (2x10^5^ PFU/ml)-infected sham or BDL operated mice were harvested 72 hpi and total RNA was isolated. Expression of (A) *Tnf-α* and (B) *Il-10* was analyzed by qPCR using specific primers. Relative gene expression was calculated by the ΔΔCt method using *Sdha* as housekeeping gene. Depicted are the mean values ± SEM from the indicated groups of mice (sham n = 5; BDL n = 4; sham-MCMV n = 9; BDL-MCMV n = 9). Statistical significance was calculated with Mann-Whitney-U-tests (***p< 0.001, **p< 0.01, *p< 0.05).

Expression levels of sham and BDL or sham-MCMV animals were compared. Furthermore, expression of sham-MCMV animals was checked against BDL-MCMV mice. As expected, MCMV infection of sham mice resulted in significantly elevated *Tnf-α* levels compared to uninfected sham mice (Fig 4A). Importantly, *Tnf-α* expression was significantly decreased in BDL-MCMV compared to sham-MCMV mice (Fig 4A). Moreover, 72 hpi, *Il-10* levels were significantly increased in sham-MCMV mice compared to sham animals (Fig 4B). In contrast, BDL mice exhibited just marginal increased *Il-10* expression comparable to sham animals. Most importantly, expression of *Il-10* in BDL-MCMV mice was further significantly elevated compared to sham-MCMV mice (Fig 4B).

Altogether, these data suggest that cholestasis diminished the MCMV-mediated induction of specific pro-inflammatory chemokines/cytokines and enhanced the expression of the anti-inflammatory interleukin IL-10 in the liver.

### BDL modifies MCMV-induced chemokine/cytokine secretion in plasma

**Fig 5:**
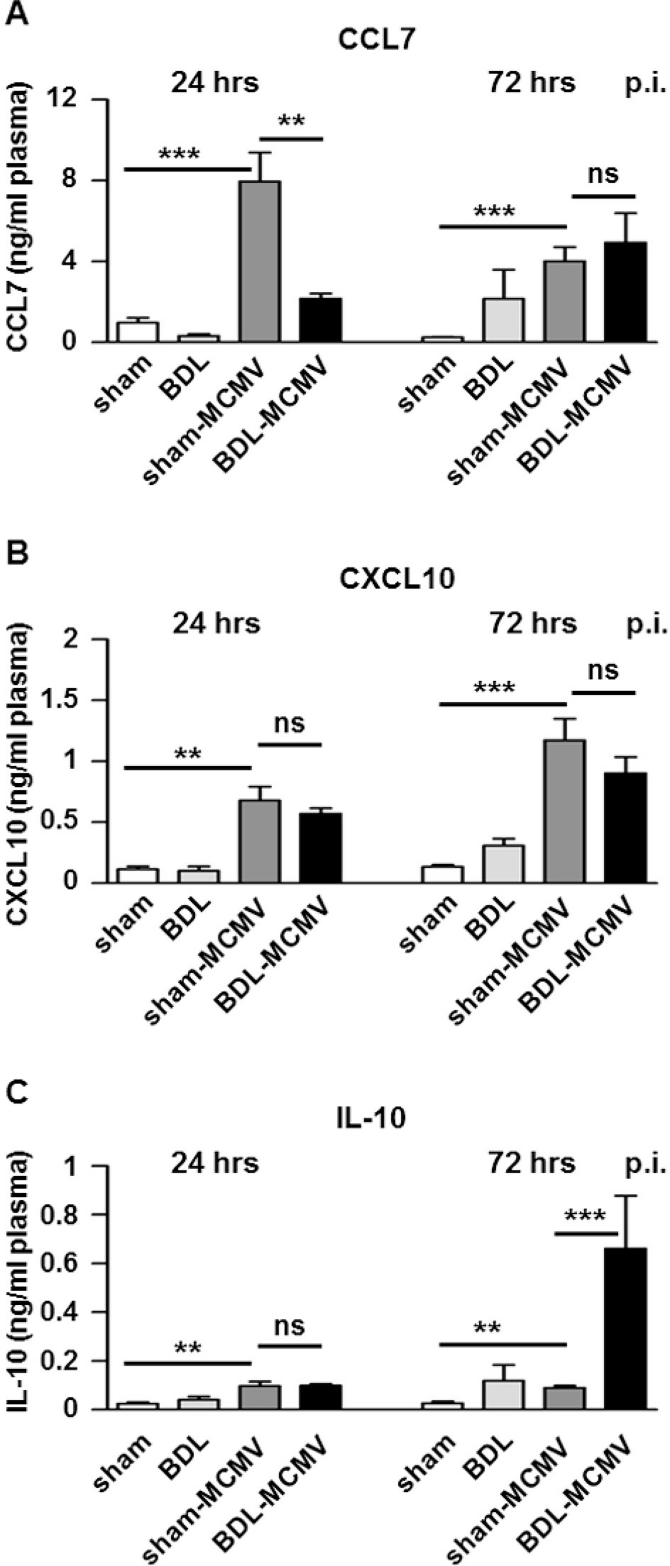
BDL modifies plasma chemokine and cytokine levels. Blood of sham- or BDL-operated animals that were either mock or MCMV-luc (2x10^5^ PFU/ml) infected was collected 24 and 72 hpi. Plasma protein levels of (A) CCL7, (B) CXCL10 or (C) IL-10 were measured using a magnetic screening assay. Depicted are the mean values ± SEM from the indicated mouse groups (24 hpi: sham n = 8; BDL n = 10; sham-MCMV n = 10; BDL-MCMV n = 10; 72 hpi: sham n = 5; BDL n = 4; sham-MCMV n = 9; BDL-MCMV n = 9). Statistical significance was calculated with Mann-Whitney-U-tests (***p< 0.001, **p< 0.01, *p< 0.05, ns: not significant).

In order to analyze if the altered MCMV-dependent chemokine/cytokine expression in the cholestatic liver has also systemic impacts, the steady state levels of CCL7, CCL12, CXCL9, CXCL10, TNF-α and IL-10 were determined in mouse plasma by a magnetic screening assay 24 and 72 hpi. Statistical significance was evaluated by Kruskal-Wallis tests. Secretion of CCL7 and CXCL10 was induced in sham-MCMV compared to sham mice at both time points analyzed. CCL7 plasma levels were significantly lower in BDL-MCMV than in sham-MCMV animals 24 hpi (Fig 5A). Comparable results were obtained investigating CCL12 (S3 Fig). In contrast, plasma levels of CXCL10 (Fig 5B) and CXCL9 (S3 Fig) in sham-MCMV and BDL-MCMV mice were comparable at both time points. Plasma TNF-α amounts remained at background levels at any condition tested (S3 Fig). However, IL-10 secretion was significantly elevated in sham-MCMV mice compared to sham mice. Furthermore, IL-10 secretion was significantly elevated in BDL-MCMV mice 72 hpi compared to sham-MCMV animals (Fig 5C). Altogether, the plasma levels of cytokines and chemokines were not strictly correlated to the corresponding mRNA levels in the liver, however, one has to take in account that immune activation in other tissues contributes to final plasma concentrations.

Taken together, these results imply a systemic diminishment of certain MCMV-induced CCR2 ligands (CCL7 and CCL12) and enhanced IL-10 secretion under cholestatic conditions, pointing to a general immuno-suppressive effect of BDL in MCMV-infected mice.

### BDL is not associated with altered MCMV replication

**Fig 6:**
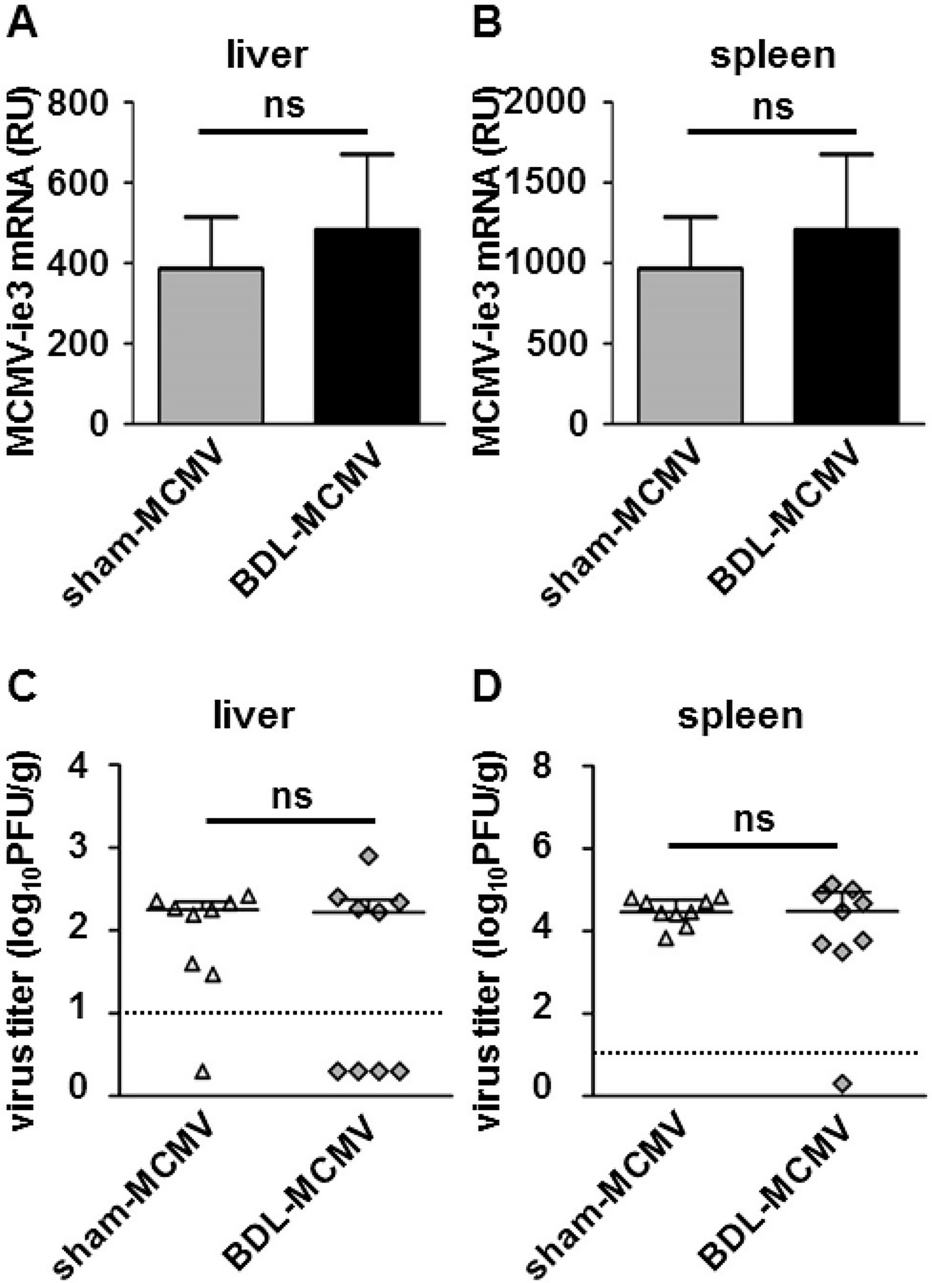
BDL is not associated with diminished MCMV replication. Sham or BDL operated mice were infected with MCMV-luc (2x10^5^ PFU/ml) and 72 hpi organs were harvested. Total RNA of liver or spleen tissue was prepared and MCMV *ie3* mRNA expression was analyzed by qPCR using specific primers or TaqMan primers and probes. Relative gene expression was calculated by the ΔΔCt method using RPII or SDHA as housekeeping gene. Depicted are the mean values ± SEM of (A) liver tissue and (B) spleen tissue 72 hpi from the indicated groups of mice (sham-MCMV n = 9; BDL-MCMV n = 9). Statistical significance was calculated with Mann-Whitney-U-tests. Organ homogenates of (C) livers or (D) spleens were generated 72 hpi and virus-tissue suspensions were titrated on murine fibroblasts. 24 h post titration luciferase activity was measured and virus titers were calculated using a standard curve. Each square represents one sample, the black line illustrates the median of each group and the dotted line displays the detection limit. Statistical significance was calculated with Mann-Whitney-U-tests (ns: not significant).

BDL-induced reduction of MCMV-dependent chemokine expression and immune cell recruitment to the liver might result in a pro-viral effect leading to enhanced virus replication in the liver. To test this assumption we determined MCMV *immediate early 3* (*ie3*) gene expression and virus titers in liver and spleen 72 hpi comparing sham- or BDL-operated mice infected with MCMV. Interestingly, significant changes of liver specific viral gene expression in sham-MCMV compared to BDL-MCMV mice were not detectable (Fig 6A).

To assess extrahepatic effects of BDL-induced cholestasis on MCMV replication we measured viral gene expression in the spleen. Again, *ie3* expression of both, sham-MCMV or BDL-MCMV infected mice were comparable (Fig 6B). Finally, we determined the titers of infectious virus in liver and spleen. Neither a significant virus titer increase nor a reduction was observed in livers (Fig 6C) or spleens (Fig 6D) of MCMV-infected sham-operated mice compared to infected BDL mice. This observation indicates that the BDL-induced defects of antiviral defense are either not sufficient to modify the MCMV virus load in the liver or that they are counteracted by other bile acid-dependent mechanisms.

In summary, BDL leads to reduced macrophage as well as NK cell infiltration and modified chemokine/ cytokine expression after MCMV infection but did not result in elevated virus titers in BDL mice.

## Discussion

In the present study, we investigated for the first time the reciprocal impact of cholestasis on virus replication and antiviral immune induction and vice versa. For this purpose we examined MCMV-infected BDL mice with respect to liver damage, immune activation and virus clearance. BDL mice exhibited largely increased bile acid levels and a bile acid composition shifting to taurine-conjugated bile acids as documented in earlier reports [36]. Our chemokine profiling analysis revealed that a particular group of chemokines including CXCL2, CXCL14 and CCL17 (Fig 3 and S1 Fig) was exclusively induced by BDL whereas CCL2, CCL7 and CXCL10 were induced by both, BDL and MCMV infection. Induction of CCL2, CXCL2 and CXCL10 by BDL was described previously [37,38]. In general, our study confirms that BDL-induced expression of several chemokines contributing to liver injury (reviewed in [39,40]). Consequently, we found elevated expression of NKT cell (PZLF) and neutrophil (CD11b) markers in the livers of BDL mice fitting earlier reports demonstrating the recruitment of these cell types to the BDL-injured liver [41,42]. Neither expression of BDL-dependent immune cell markers (Fig 1) nor production of BDL-induced chemokines was further regulated by MCMV replication in the liver (Fig 3) indicating that MCMV infection does not aggravate liver damage which fits to our clinical data (Table 1).

MCMV infection causes a pronounced activation of the chemokine system in the liver. In accordance with previous reports we observed a strong induction of the CC-chemokine CCL2 [28] and the CXC-chemokines CXCL9 and CXCL10 [29]. Additionally, we found enhanced induction of CCL4, CCL7, CCL8 and CCL12 (Fig 3 and S1 Fig). Whereas CCL7 and CCL8 had been reported to be induced in HCMV-infected macrophages [43], CCL12 has not been linked to CMV infection so far. The major inflammatory cells recruited to the MCMV-infected liver are Ly6C^hi^ monocytes [24], F4/80^+^/CD11b^+^ macrophages and NK1.1^+^ NK cells [25] which is in line with our observations (Fig 1). Importantly, our investigation of MCMV-infected BDL mice indicated severe perturbations in MCMV triggered recruitment of immune cells (Fig 1) as well as in expression of CCL4, CCL7, CCL8, CCL12, CXCL9 and CXCL10, which were clearly reduced in MCMV-infected BDL mice (Fig 3 and S1 Fig). Analyzing the plasma levels of MCMV-induced chemokines we found reduced amounts of CCL7 (Fig 5) and CCL12 (S3 Fig)in plasma of BDL-MCMV animals whereas CCL2, CXCL9 and CXCL10 (Fig 5 and S3 Fig) were comparable in sham- and BDL-operated infected mice. Hence, the systematic relevance of BDL-mediated modification of chemokine induction appears limited to distinct CCR2 ligands. BDL disturbs expression of CCL7 and CCL12, but not of CCL2, which is believed to be the major mediator of MCMV-induced cell migration [28] although a contribution of CCL7 and CCL12 to the regulation of macrophage recruitment from bone marrow during MCMV infection has also been described [44]. Importantly, there is growing evidence that CCL7 and CCL2 functions are not simply redundant. CCL2 and CCL7 are both implicated in monocyte recruitment during bacterial infection with *Listeria monocytogenes* [45]. Immune responses against the parasite *Leishmania major* uncovered a CCL2-independent role of CCR2 [46]. Furthermore, CCL2 and CCL7 exhibit differential functions during infections with West Nile Virus whereby CCL7 is the critical chemokine for virus clearance and survival [47]. Hence, we conclude that the BDL-mediated disturbance of CCR2 ligand expression might be responsible for our observation of reduced immigration of F4/80^+^ cells to the MCMV-infected liver (Fig 2).

NK cells activated during MCMV infection are reported to be regulated via CCL3, which was not significantly affected by BDL [48]. However, four receptors appear to play a key role in mouse NK cell recruitment following an inflammatory stimulus: CCR2, CCR5, CXCR3 and CX3CR1 [30]. These receptors allow NK cells to respond to a large array of inflammatory chemokines such as CCL2, CCL3, CCL5, CCL7, CCL8, CCL9, CCL11, CCL13, CXCL9-11 and CX3CL1. Thus, NK cell recruitment in the same tissue can be mediated by different chemokine receptors [30] Besides CCR5, CCR2 has been reported to be important for NK cell recruitment to the liver of (MCMV)-infected mice [28]. A further candidate is CXCR3 whose ligands CXCL9 and CXCL10 were clearly reduced in MCMV-infected BDL mice (Fig 3 and S1 Fig). A role for CXCR3 in NK cell recruitment during MCMV infection has not been established so far. However, reduced numbers of intrahepatic NK cells upon CXCL10 neutralization have been detected in dengue virus-infected mice indicating that CXCL10 is critical for recruiting activated NK cells to sites of dengue infection [49].

At first glance, reduced immune cell infiltrate and perturbation of antiviral chemokine induction during cholestasis should be detrimental for the host and result in enhanced viral replication, which is not observed in our study whereas infected CCR2^-/-^ mice exhibited enhanced MCMV replication in liver and spleen [28]. However, according to current knowledge, the diminished recruitment of NK cells to the MCMV-infected liver would be the only clear antiviral mechanism affected by BDL and most likely result in enhanced virus replication. In contrast, there are three other BDL-associated findings possibly contributing to a virus yield reduction. Firstly, BDL reduced the recruitment of Ly6C^hi^ inflammatory monocytes which have been demonstrated to lack antiviral activity. In contrast, Ly6C^hi^ monocytes modulate antiviral immunity by reducing the capacity of T cells to eliminate virus-infected cells [24]. Furthermore, immunosuppressive properties of Ly6C^hi^ monocytes have been shown to delay virus clearance in the adenoviral hepatitis [50] model underlining a pro-viral role of this cell type. Conversely, their reduced recruitment under BDL could have an overall antiviral effect. Secondly, we were able to detect effective concentrations of potentially antiviral bile acids in BDL and BDL-MCMV mice (Table 1). We recently demonstrated anti-cytomegalovirus properties of the hydrophobic bile acids TCDC, glycochenodeoxycholic acid, and to a lesser extent TC *in vitro* [51]. Hence, a direct inhibition of virus replication in the liver by the accumulation of bile acids during BDL might contribute to the limitation of MCMV replication in the liver. Thirdly, a recent study demonstrated a direct activity of neutrophils as TRAIL-dependent antiviral effectors against MCMV [52]. Since neutrophils are directly recruited by BDL, their presence in the liver could provide an additional antiviral potential. Additionally, it has to be taken in account that the liver contains substantial numbers of innate lymphoid cells (ILCs). Group 1 ILCs share phenotypic and functional similarity with NK cells [53] and might thereby maintain basic antiviral functions within the liver in the absence of additional NK cell recruitment to the liver. At the current state, we were not able to define the relative contribution of ILC and NK cells since gene expression profiles cannot clearly distinguish those cell types. Hence, flow cytometric studies are needed to to further determine number and activation state of ILC and NK cells under the conditions of BDL and MCMV-infection. Overall, the impact of BDL-mediated immune alterations on MCMV replication was unexpectedly low. However, replication of other hepatotropic viruses like mouse hepatitis virus (MHV) may exhibit a different pattern since direct involvement of the BDL-downregulated chemokines CXCL9 and CXCL10 in anti-MHV defense has already been shown, highlighting that host- virus interactions are not generalizable, even though the liver tissue provides a common environment as starting point for virus infection [54,55].

Importantly, our findings might have an even larger impact for the understanding of the interplay between cholestasis and bacterial infections. The cells most strongly affected by BDL dysregulation appeared to be Ly6C^hi^ monocytes (Fig 1). This cell type is not described to contribute to antiviral defense, while pro-viral properties of Ly6C^hi^ monocytes have been clearly demonstrated [24,50]. However, they play a central role in the control of gram-positive bacteria and are important for the successful control of *Listeria monocytogenes* infections [56]. Of note, an impaired clearance of *Listeria monocytogenes* from the liver of infected BDL-operated mice has already been described [21]. Likewise, BDL mice showed a deteriorated resistance to *Escherichia coli* infection exhibiting a higher mortality rate and increased bacterial growth compared to sham-operated animals [12]. Additionally, BDL mice infected with *E*. *coli* generate higher IL-10 expression levels than sham-operated littermates [12]. IL-10 has a pronounced anti-inflammatory function and negatively impacts macrophage function and expression of pro-inflammatory cytokines. However, a causative role of IL-10 for impaired bacterial clearance after BDL could not be proven [27]. We also observed strongly increased levels of IL-10 in liver tissue (Fig 4) and plasma (Fig 5) in response to viral infection. However, it appears unlikely that the MCMV-induced chemokine induction is solely suppressed due to enhanced IL-10 secretion after BDL since it was not detectable 24 hpi (Fig 5) where chemokine expression was already impaired (S2 Fig). Therefore, we assume that the elevated IL-10 levels observed in both, MCMV- und *E*.*coli*-infected BDL mice are rather a consequence then the cause of reduced chemokine expression and immune cell recruitment.

In conclusion, our data point to a common pathogenic mechanism that is based on a general defect of chemokine induction in response to pathogens and is caused by BDL-induced cholestasis. Under cholestasis the expression of a specific chemokine subset in the liver is reduced mainly affecting the CCR2 and CXCR3 axes of immune cell trafficking. Finally, this defect reduces monocyte and NK cell recruitment to the liver. We have recently demonstrated that bile acids are able to induce an anti-inflammatory phenotype in human macrophages [57]. Importantly, bile acid treatment of human macrophages reverts several pro-inflammatory effects of LPS which are also observed in this study including TNF-α induction (Fig 4) as well as induction of chemokines involved in CCR2- and CXCR3-dependent signaling (Fig 3). These results make Kupffer cells in the liver to be the most promising candidate to be affected by bile acid accumulation. A comparable reprogramming of Kupffer cells could result in an anti-inflammatory phenotype responsible for a general defect in chemokine induction. Of note, IL-10 was not directly affected by bile acids. Remarkably, infiltrating CD11b^+^Ly6G^+^ neutrophils have been identified as the main producers of IL-10 during systematic bacterial infections [58]. Since accumulation of neutrophils after BDL is well-known [22] and our results indicate that MCMV infection does not impact recruitment of CD11b^+^ cells in infected BDL mice (Fig 1B) it is tempting to speculate that reduced chemokine levels and increased IL-10 levels are not only temporarily distinct events but are also induced by different cellular sources of the respective cytokine.

Based on our findings and the current knowledge, we propose a biphasic pathogenesis model of reduced immune activation after cholestasis. In the first phase bile acids might act as signaling molecules which limit the induction of pro-inflammatory chemokines (most probably by Kupffer cells) in the liver resulting in a diminished capacity to recruit inflammatory macrophages and NK cells to the liver. This might be a disadvantage during pathogen infections but has the clear advantage to limit excessive inflammatory tissue damage by cholestasis. In the second phase neutrophils recruited by BDL respond to the respective pathogens by IL-10 production and thereby further augment the anti-inflammatory environment established by cholestasis. Further studies are needed to prove this concept and to identify the cell types within the liver which initiate the observed alterations of chemokine induction. Based on the findings of this study multi-color FACS analysis should be used to determine the individual contributions of Kupffer cells, ILCs, liver macrophages, neutrophils or hepatocytes to virus-induced immune activation and its modulation by cholestasis.

This concept has also to be proven in other infection models of the liver and may provide new options for prevention and therapy of infections in cholestatic patients.

## Methods

### Analysis of liver function

Glutamate pyruvate transaminase (GPT) levels were measured in blood plasma of mice using a dry clinical chemistry analyzer (Spotchem EZ-SP-4410, arkray, Amstelveen, Netherlands) and GPT-test strips. Bile acids were quantified in mouse plasma by ultra-high performance liquid chromatography tandem mass spectrometry (Waters, UK) [59].

### Mice and bile duct ligation

Male C57BL/6 were purchased from Janvier Labs (France) and maintained in the animal facility of the Universtity of Duesseldorf (ZETT, Duesseldorf, Germany). Mice were sham- or BDL-operated and afterwards either mock treated (PBS injection) or infected intraperitoneally with Δm157-MCMV-luciferase (MCMV-luc, 2x10^5^ PFU). Mice were anesthetized with isoflurane (1.5–2%) before the *ductus hepatocholedochus* was isolated and ligated. Sham-operated mice underwent the same procedure without bile duct ligation. After 24 or 72 h mice were anesthetized with Ketamine (100 mg/kg body weight) and Xylazine (20 mg/kg body weight), blood was collected from the *vena cava* and plasma was separated using Microtainer EDTA tubes (BD Biosciences). Livers and spleens were harvested and stored in liquid nitrogen. Animal care and experiments were performed according to the German law for animal protection. All animal work was approved by the Landesamt fuer Natur, Umwelt und Verbraucherschutz (North-Rhine Westfalia, Germany), file number AZ 84–02.04.2011.A201.

### Virus infection and examination of tissue virus titers

MCMV-Δm157-luc was constructed based on the MCMV-BAC pSM3fr-MCK-2fl [60] (kindly provided by B. Adler, Munich, Germany). Deletion of the *m157* ORF and insertion of the luc sequence into the *m157* locus were performed exactly as described [61]. To quantify organ virus titers, organs were homogenized and virus-tissue suspensions were titrated on a stable mouse embryonic fibroblast cell line. 24 h post titration cells were lysed in luciferase buffer (Roche) and luciferase activity was measured (TriStar^2^; Berthold Technologies). Virus titer was calculated utilizing a virus standard curve.

### Polymerase chain reaction

RNA was isolated following manufacturer´s instructions (RNeasy Mini Kit Qiagen, Hilden, Germany) before cDNA was synthesized as described (QuantiTect Kit, Qiagen). Quantitative PCR (qPCR) was carried out on a ViiA7 real-time-PCR System (Applied Biosystems, Foster City, CA) using SYBR Green as reporter dye (Promega, Mannheim, Germany) or TaqMan assays (TaqMan Universal PCR Master Mix, Thermo Fisher Scientific, Darmstadt, Germany). Results were calculated by the ΔΔCt method using RNA polymerase II (RPII), succinate dehydrogenase (sdha) or actin beta (Actb) as housekeeping genes for normalization. The following primer sequences were used for TaqMan-PCR with SYBRGreen detection:

Tnf-α for: gctgagctcaaaccctggta; Tnf-α rev: cggactccgcaaagtctaag;

Il-10 for: ccaagccttatcggaaatga; Il-10 rev: tcctgagggtcttcagcttc;

Sdha for: tggggagtgccgtggtgtca; Sdha rev: gtgccgtcccctgtgctggt;

Actb for: ttgctgacaggatgcagaag; Actb rev: tgatccacatctgctggaag;

F4/80 for: cctgtcaaccaggctttgtc; F4/80 rev: gagagtgttgtggcaggttg;

CD335 for: gccagaggatcaacactgaa; CD335 rev: accgagtttccatttgtgacc;

CD69 for: tcacatctggagagagggca; CD69 rev: aacacagcccaagggatagaa;

CXCL1 for: caagaacatccagagcttgaaggt; CXCL1 rev: gtggctatgacttcggtttgg;

CXCL9 for: tgcacgatgctcctgca; CXCL9 rev: aggtctttgagggatttgtagtgg;

CCL2 for: gctggagcatccacgtgtt; CCL2 rev: atcttgctggtgaatgagtagca.

For TaqMan-PCR with FAM-TAMRA the following primer/probe combinations were used:

AZ-mNK1.1-forw: TTGCTGCTCATTCAAGACC

AZ-mNK1.1-rev: TTGTGCCATTTATCCACTTCC

AZ-mNK1.1-probe: GGATTGGACTAAGGTTCACATTGCCAGA

AZ-mCD11b-forw: TCACCTTCATCAACACAACC

AZ-mCD11b-rev: CCAAGCCAATATGCTGATACC

AZ-mCD11b-probe: GCAGTCATCTTGAGGAACCGTGTCCAAA

AZ-mLy6C-forw: CATCTGACAGAACTTGCCAC

AZ-mLy6C-rev: GAAGAATGAGCACACAGGAC

AZ-mLy6C-probe: TGAGAGGAACCCTTCTCTGAGGATGGAC

AZ-mPLZF-forw: TCATTCAGCGGGTGCCAAAG

AZ-mPLZF-rev: TCCCACACAGCAGACAGAAG

AZ-mPLZF-probe: CTTTGTGTGTGATCAATGCGGTGCCCAGT

AZ-mCMVie3-forw: CATGTCGCCAACAAGATCC

AZ- mCMVie3-rev: CGCTGCTGTAACAATATCTATGTTC

AZ-mCMVie3-probe: AGAGAAGATCCTCATGGACCGCATCGCTGAC

AZ-mRPII–forw: CGCACCACGTCCAATGATA

AZ-mRPII–rev: TGTGCTGCTGCTTCCATAAG

AZ-mRPII-probe: CTGTACCATGTCATCTCCTTTGATGGTTCTTATG

Commercial TaqMan Assays were purchased by Thermo Fisher Scientific (Darmstadt, Germany) for CXCL2 (00436450), CXCL10 (00445235), CXCL12 (00445552), CXCL14 (00444699), CX3CL1 (00436454), CCL3 (00441258), CCL4 (00443111), CCL5 (01302427), CCL7 (00443113), CCL8 (01297183), CCL9 (00441260), CCL11 (00441238), CCL12 (01617100), CCL17 (00516136).

### Detection of blood chemokine/cytokine levels

Chemokine/cytokine levels in blood plasma were analyzed with a magnetic screening assay kit (RandD systems, Minneapolis, USA) on a Luminex 200 Analyzer (Luminex, s´Hertogenbosch, Netherlands) following the manufacturers’ protocol. Samples were diluted with PBS.

### Immunohistochemistry

Tissue sections from cryo-conserved liver were fixed with methanol and blocked with PBS/5% BSA. Thereafter tissue sections were incubated with F4/80 (AbD Serotec, Oxford, UK) antibodies (Optistain/Dianova, Suffolk, UK). Anti-rat Alexa Fluor 488-conjugated antibody (Jackson ImmunoResearch/Dianova, Hamburg, Germany) was used as secondary antibody and nuclei were stained with DAPI. Brightness and contrast were adjusted in the same way for all pictures in Fig 2A.

### Statistical analysis

Data are depicted as the mean ± standard error of the mean (SEM). Statistical calculations were done using GraphPad Prism software version 5.04. Because of the small sample size and the non-normally distributed data Kruskal-Wallis Tests were performed to determine statistical significance. If this test was statistically significant, for the whole data set p-values were calculated by Mann-Whitney-U-tests (two-sited). 24 hpi: sham n = 8; BDL n = 10; sham MCMV n = 10; BDL MCMV n = 10; 72 hpi: sham n = 5; BDL n = 4; sham MCMV n = 9; BDL MCMV n = 9. For statistical analysis all samples were considered.

## Supporting information

S1 Fig Modification of liver chemokine expression by BDL or MCMV 72 h after treatment. Sham- or BDL-operated mice were either mock treated or infected with MCMV-luc (2x10^5^ PFU/ml) and 72 hpi organs were harvested to prepare total RNA of liver tissue. Chemokine mRNA expression was analysed by qPCR using specific primers or TaqMan primers and probes (sham n = 5; BDL n = 4; sham-MCMV n = 9; BDL-MCMV n = 9). Depicted are chemokines analyzed which are not shown in Fig 3. *Ccl11* and *Cx3cl1* were included in the chemokine panel but were not affected by any condition.
(PPTX)

S2 Fig Modification of liver chemokine expression by BDL or MCMV 24 h after treatment. Sham- or BDL-operated mice were either mock treated or infected with MCMV-luc (2x10^5^ PFU/ml) and 24 hpi organs were harvested to prepare total RNA of liver tissue. Chemokine mRNA expression was analyzed by qPCR using specific primers or TaqMan primers and probes (sham n = 8; BDL n = 10; sham-MCMV n = 10; BDL-MCMV n = 10). Depicted are chemokines analyzed 24 h after treatment which were affected either by BDL or MCMV-infection.
(PPTX)

S3 Fig Modification of plasma chemokine and cytokine levels by BDL or MCMV 24 h and 72 h after treatment. Blood of sham-or BDL-operated animals that were either mock or MCMV-luc (2x10^5^ PFU/ml) infected was collected 24 and 72 hpi. Plasma protein levels of (A) CCL12, (B) CXCL9 or (C) TNF-α were measured using a magnetic screening assay. Depicted are the mean values ± SEM from the indicated mouse groups (24 hpi: sham n = 8; BDL n = 10; sham-MCMV n = 10; BDL-MCMV n = 10; 72 hpi: sham n = 5; BDL n = 4; sham-MCMV n = 9; BDL-MCMV n = 9). Statistical significance was calculated with Mann-Whitney-U-tests (***p< 0.001, **p< 0.01, *p< 0.05, ns: not significant).
(PPTX)
