# Supplementary material for: Anti-inflammatory consequences of bile acid accumulation in virus-infected bile duct ligated mice
Source: PLoS One. 2018 Jun 28;13(6):e0199863. doi: 10.1371/journal.pone.0199863 (PMC6023182; doi:10.1371/journal.pone.0199863)
Supplement: S1 Fig — Sham- or BDL-operated mice were either mock treated or infected with MCMV-luc (2x10^5^ PFU/ml) and 72 hpi organs were harvested to prepare total RNA of liver tissue. Chemokine mRNA expression was analysed by qPCR using specific primers or TaqMan primers and probes (sham n = 5; BDL n = 4; sham-MCMV n = 9; BDL-MCMV n = 9). Depicted are chemokines analyzed which are not shown in Fig 3. Ccl11 and Cx3cl1 were included in the chemokine panel but were not affected by any condition. (PPTX) [file pone.0199863.s001.pptx]

## Slide 1
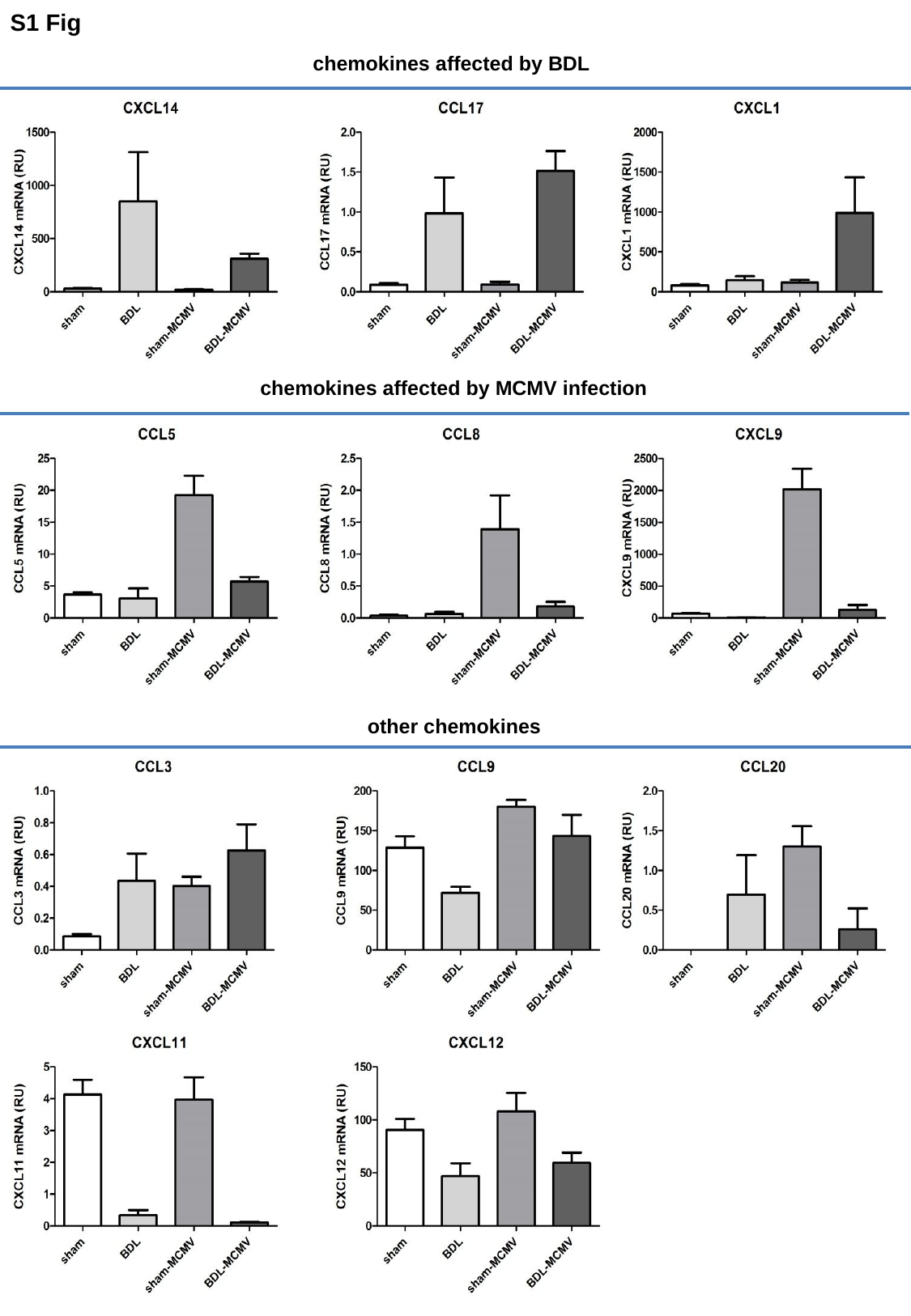

S1 Fig
chemokines affected by BDL
chemokines affected by MCMV infection
other chemokines
