# Supplementary figures and images for: Anti-inflammatory consequences of bile acid accumulation in virus-infected bile duct ligated mice
Source: PLoS One. 2018 Jun 28;13(6):e0199863. doi: 10.1371/journal.pone.0199863 (PMC6023182; doi:10.1371/journal.pone.0199863)

## Slide 1
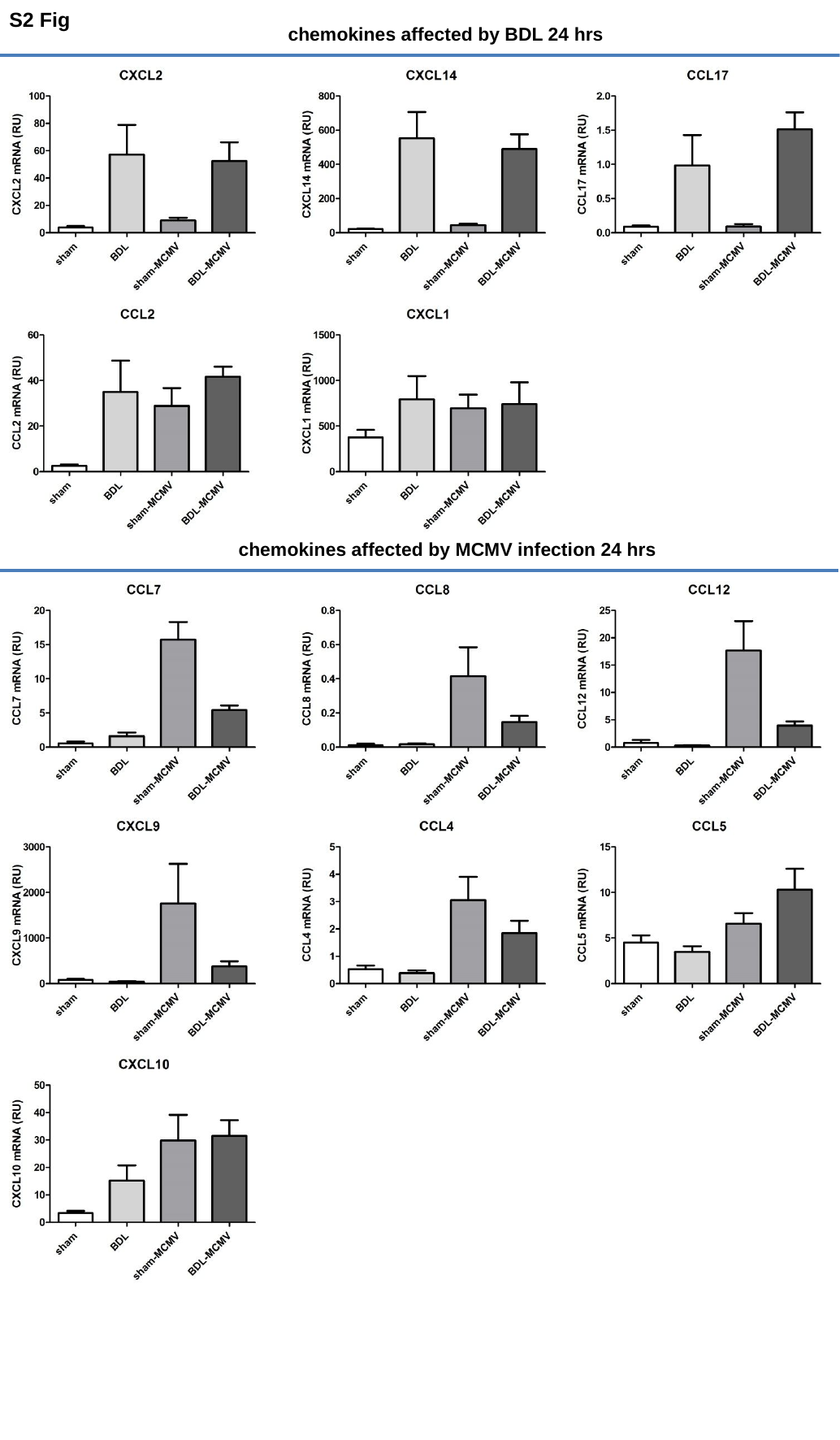

S2 Fig
chemokines affected by BDL 24 hrs
chemokines affected by MCMV infection 24 hrs

Supplement: S2 Fig — Sham- or BDL-operated mice were either mock treated or infected with MCMV-luc (2x10^5^ PFU/ml) and 24 hpi organs were harvested to prepare total RNA of liver tissue. Chemokine mRNA expression was analyzed by qPCR using specific primers or TaqMan primers and probes (sham n = 8; BDL n = 10; sham-MCMV n = 10; BDL-MCMV n = 10). Depicted are chemokines analyzed 24 h after treatment which were affected either by BDL or MCMV-infection. (PPTX) [file pone.0199863.s002.pptx]
