# Supplementary material for: Anti-inflammatory consequences of bile acid accumulation in virus-infected bile duct ligated mice
Source: PLoS One. 2018 Jun 28;13(6):e0199863. doi: 10.1371/journal.pone.0199863 (PMC6023182; doi:10.1371/journal.pone.0199863)
Supplement: S3 Fig — Blood of sham-or BDL-operated animals that were either mock or MCMV-luc (2x10^5^ PFU/ml) infected was collected 24 and 72 hpi. Plasma protein levels of (A) CCL12, (B) CXCL9 or (C) TNF-α were measured using a magnetic screening assay. Depicted are the mean values ± SEM from the indicated mouse groups (24 hpi: sham n = 8; BDL n = 10; sham-MCMV n = 10; BDL-MCMV n = 10; 72 hpi: sham n = 5; BDL n = 4; sham-MCMV n = 9; BDL-MCMV n = 9). Statistical significance was calculated with Mann-Whitney-U-tests (***p< 0.001, **p< 0.01, *p< 0.05, ns: not significant). (PPTX) [file pone.0199863.s003.pptx]

## Slide 1
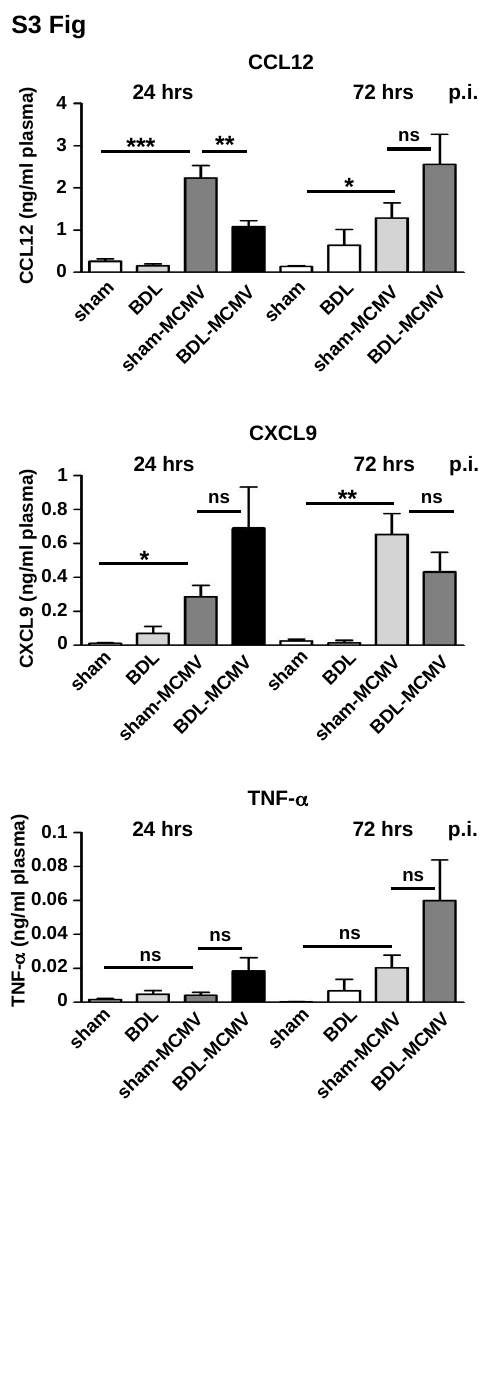

S3 Fig
CCL12
24 hrs
72 hrs p.i.
4
ns
**
***
3
*
CCL12 (ng/ml plasma)
2
1
0
BDL
sham
BDL-MCMV
sham-MCMV
BDL
sham
BDL-MCMV
sham-MCMV
CXCL9
24 hrs
72 hrs p.i.
1
**
ns
ns
0.8
0.6
*
CXCL9 (ng/ml plasma)
0.4
0.2
0
BDL
sham
BDL-MCMV
sham-MCMV
BDL
sham
BDL-MCMV
sham-MCMV
TNF-a
24 hrs
72 hrs p.i.
0.1
0.08
0.06
0.04
0.02
0
ns
TNF-a (ng/ml plasma)
ns
ns
ns
BDL
sham
BDL-MCMV
sham-MCMV
BDL
sham
BDL-MCMV
sham-MCMV
